# Endomembrane Tension and Trafficking

**DOI:** 10.3389/fcell.2020.611326

**Published:** 2021-01-08

**Authors:** Amra Saric, Spencer A. Freeman

**Affiliations:** ^1^Neurosciences and Cellular and Structural Biology Division, Eunice Kennedy Shriver National Institute of Child Health and Human Development, National Institutes of Health, Bethesda, MD, United States; ^2^Program in Cell Biology, Peter Gilgan Center for Research and Learning, Hospital for Sick Children, Toronto, ON, Canada; ^3^Department of Biochemistry, University of Toronto, Toronto, ON, Canada

**Keywords:** endocytosis, phagocytosis, macropinocytosis, mTOR, ESCRT, ion transport, V-ATPase, sorting nexin

## Abstract

Eukaryotic cells employ diverse uptake mechanisms depending on their specialized functions. While such mechanisms vary widely in their defining criteria: scale, molecular machinery utilized, cargo selection, and cargo destination, to name a few, they all result in the internalization of extracellular solutes and fluid into membrane-bound endosomes. Upon scission from the plasma membrane, this compartment is immediately subjected to extensive remodeling which involves tubulation and vesiculation/budding of the limiting endomembrane. This is followed by a maturation process involving concomitant retrograde transport by microtubule-based motors and graded fusion with late endosomes and lysosomes, organelles that support the degradation of the internalized content. Here we review an important determinant for sorting and trafficking in early endosomes and in lysosomes; the control of tension on the endomembrane. Remodeling of endomembranes is opposed by high tension (caused by high hydrostatic pressure) and supported by the relief of tension. We describe how the timely and coordinated efflux of major solutes along the endocytic pathway affords the cell control over such tension. The channels and transporters that expel the smallest components of the ingested medium from the early endocytic fluid are described in detail as these systems are thought to enable endomembrane deformation by curvature-sensing/generating coat proteins. We also review similar considerations for the lysosome where resident hydrolases liberate building blocks from luminal macromolecules and transporters flux these organic solutes to orchestrate trafficking events. How the cell directs organellar trafficking based on the luminal contents of organelles of the endocytic pathway is not well-understood, however, we propose that the control over membrane tension by solute transport constitutes one means for this to ensue.

## Introduction

**Figure 1:**
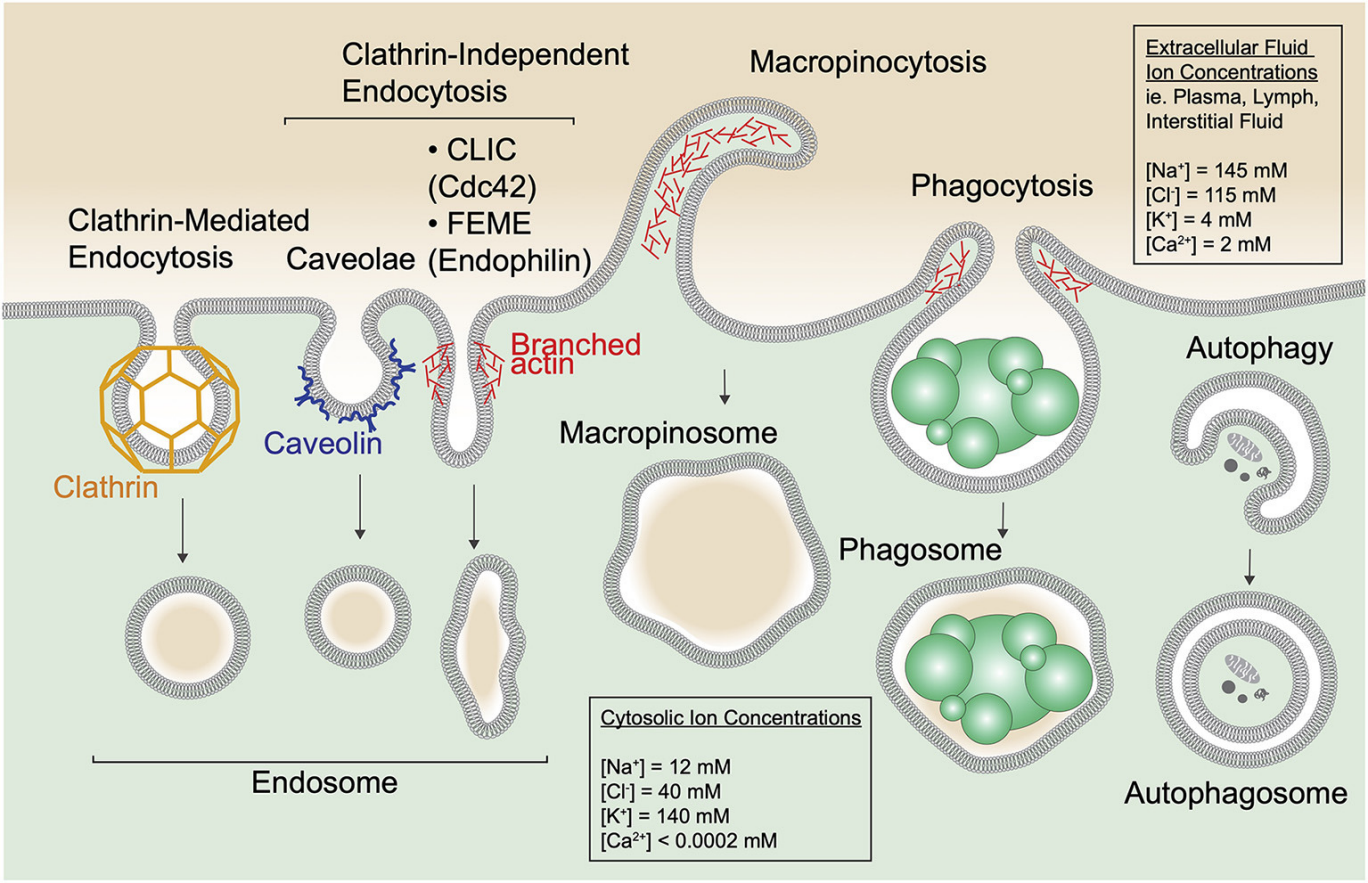
Cells entrap extracellular and cytosolic fluid via diverse mechanisms. Multiple forms of endocytic uptake are depicted along with autophagy. Clathrin-mediated endocytosis occurs when plasma membrane (PM) invaginations are formed by the coat protein clathrin to generate a clathrin-coated pit. The pit dissociates from the PM by scission, induced by the GTPase dynamin (not depicted), and forms an endocytic vesicle. In addition, numerous clathrin-independent endocytic routes are depicted including caveolae, clathrin-independent carriers (CLICs), and fast endophilin-mediated endocytosis (FEME). Caveolae form at cholesterol-rich PM domains called lipid rafts and use the protein caveolin to shape the membrane into flask-like invaginations. CLICs are uncoated tubulovesicular PM invaginations stabilized by the actin cytoskeleton and regulated by the small GTPase Cdc42 that recruits the actin polymerization machinery. FEME relies on a BAR-domain containing protein, endophilin, for curvature of tubular PM invaginations that scission upon dynamin recruitment. In the schematic, one tubular PM invagination represents endocytic uptake via CLICs and FEME. Macropinocytosis and phagocytosis are specialized forms of endocytosis. Macropinocytosis proceeds via polymerization of the cortical actin cytoskeleton to produce PM ruffles that fold back on the cell and fuse, indiscriminantly trapping the surrounding medium into large (up to 5 μm sized) membrane-enclosed vacuoles called macropinososmes. Phagocytosis is a receptor-mediated process by which cells bind and engulf particulates including dead cells (depicted), debris, and pathogens like bacteria and fungi into membrane-enclosed phagosomes. Phagocytosis is also aided by the actin cytoskeleton. Note that extracellular fluid is internalized via all these pathways. In addition, cells use autophagy to sequester protein aggregates, damaged organelles and other cytoplasmic components within a double-membrane structure called an autophagosome.

The active internalization of extracellular material by eukaryotic cells is key to nutrient acquisition, environment sensing, and maintenance of normal cell physiology. In metazoans, this process is essential for maintaining the specialized functions of tissues and the system as a whole. As such, different cell types engage multiple different uptake pathways including clathrin-dependent and -independent endocytosis, micropinocytosis, and phagocytosis (Figure 1). These pathways operate under distinct mechanisms and scales; receptor-mediated endocytosis internalizes small plasma membrane-derived vesicles, macropinocytosis results in bulk uptake of extracellular fluid, and phagocytosis is employed for the internalization of large (>0.5 μm) particulates. Endocytic pathways are also usurped by a variety of obligate intracellular pathogens as part of their infection cycle including viruses, bacteria, and fungi. In addition, cells undergo autophagy, a process by which cytosolic components are entrapped within a newly generated membrane to form an autophagosome. Despite their differences, these pathways all result in the conception of an intracellular, membrane-bound vesicle bearing cargo (i.e., receptors, ligands), and fluid.

Cargo that is internalized from the extracellular milieu meets one of 3 known fates depending on the cell's needs; (1) receptors/ligands may be recycled back to the plasma membrane, (2) cargo may be routed elsewhere in the cell such as to the *trans*-Golgi network (TGN) via retrograde transport or (3) cargo may be degraded by delivery to lysosomes, organelles that support the enzymatic breakdown of macromolecules (Lawrence and Zoncu, 2019). Regardless of destination, cargo sorting and trafficking from the early (nascent) endosome necessitates an astonishing degree of endomembrane remodeling. This remodeling begins moments after scission of the endosome from the plasma membrane. To retrieve receptors and membrane, fine membrane tubules extend from the endosome and pinch off to form smaller vesicles that recycle the cargo (Yamashiro et al., 1984; Ren et al., 1998; Grant and Donaldson, 2009). These fission events occur even as endosomes undergo homotypic fusion giving rise to a complex balance between membrane addition and removal. After the initial stages of remodeling, the remaining endosome that bears cargo destined for degradation then matures. Maturation can be envisaged as a series of steps including retrograde transport of the organelle, inward budding of the limiting endomembrane, morphing into a multivesicular endosome in the process (Gruenberg, 2020), and finally, graded fusion with (endo)lysosomes (Huotari and Helenius, 2011). Interestingly, these trafficking pathways may be highjacked or arrested by effectors generated by internalized pathogens. For example, bacterial effectors, ejected into the cytosol from their resident vacuoles, can target various steps of endosome maturation, thereby curtailing microbicidal activities of the host (Gruenberg and van der Goot, 2006). Studies on cargo sorting have revealed key protein complexes such as retromer, retriever, and ESCRT that function in the aforementioned pathways of retrograde cargo transport to the TGN, recycling to the plasma membrane and degradation in lysosomes, respectively (Seaman et al., 1998; Raiborg and Stenmark, 2009; McNally et al., 2017).

On the other hand, the fate of the internalized fluid has been relatively unexplored. Recent work suggests that the resolution of volume from the endocytic pathway is not only essential for sorting and trafficking, but may be the initiating event that enables the extensive endomembrane remodeling described above (Freeman et al., 2020). One need only consider the extreme surface-to-volume changes that occur during sorting (Freeman and Grinstein, 2018): A newly formed spherical macropinosome that is anywhere between 0.5 and 5 μm in diameter can project numerous fine tubules that can be several microns long yet contain almost no luminal volume at all (Kerr et al., 2006; Freeman et al., 2020). Such continuous removal of membrane without a parallel loss of volume would quickly limit this system by increasing the hydrostatic pressure within the vacuole, generating a turgid membrane that is refractory to deformation. The solution? Cells rely on a collection of vacuolar channels and transporters for the timely release of solutes from the lumen across the endomembrane. This process ensures that water is forcibly extruded from the vacuole, causing a subsequent drop in its internal hydrostatic pressure and a drop in the membrane tension. The membrane slack afforded by this process results in crenation that permits the recruitment and assembly of curvature sensing/stabilizing proteins, such as BAR domain-containing proteins, to the vacuolar surface (Simunovic and Voth, 2015; Freeman et al., 2020). It is expected therefore that trafficking complexes that associate with highly curved membranes are similarly dependent on membrane tension relief for their recruitment and function. Conversely, the addition of tension to the membrane may be utilized to arrest endomembrane remodeling. Thus, the flux of solutes, and as a consequence, water, to and from the vacuole drives traffic by controlling membrane tension.

Although tremendously understudied, similar mechanisms of membrane tension control may operate at the late stages of endosome maturation as well. As endosomes mature, they ultimately fuse with lysosomes, highly acidic organelles that harbor more than 50 resident acid hydrolases that support the degradation of internalized molecules. And, in addition to endocytosis, autophagy converges on the lysosome for degradation of intracellular cargoes through fusion of autophagosomes with lysosomes to form autolysosomes. While acidification is central to lysosomal function, it comes with osmotic considerations. For example, counter-ion fluxes that are driven by lysosome-resident transporters and channels are key to reaching and maintaining the low pH but have varying osmotic effects on the lysosome. In addition, the liberation of building blocks from enzymatic digestion of the internalized content requires their efflux via transporters of the solute carrier family (SLCs), for use in anabolic cellular processes and to mitigate an osmotic burden that could result in overtly high membrane tension. Thus, the collective activities of numerous lysosomal channels and transporters supports normal lysosomal functions and affords the cell control over endomembrane tension to dictate trafficking. When solute transport mechanisms are impaired, defects in lysosomal function and trafficking ensue, such as those documented in numerous lysosomal storage disorders (LSDs). Moreover, the ability for endocytosed liposomes, exosomes, and enveloped viruses to fuse with the limiting endomembrane are also predicted to be dependent on membrane tension. Understanding how cells utilize solute transport to control endomembrane tension is therefore important to fully appreciate mechanisms that support trafficking, infection, and its dysregulation in storage disorders.

In this review we discuss how cells may be afforded control over membrane tension to regulate endomembrane trafficking, a view that we and others have proposed (Scott and Gruenberg, 2011; Freeman and Grinstein, 2018). A brief overview of the different endocytic pathways is presented with considerations of plasma membrane tension in these processes and the solute composition of internalized fluid. This is followed by a description of how nascent endosomes flux osmolytes via a suite of channels and transporters to maintain the low endomembrane tension required for the membrane remodeling that accompanies cargo sorting and trafficking. Finally, we consider how lysosomal solute transport via a broad range of channels and transporters may function to fine-tune membrane tension as a mechanism to tightly control membrane trafficking.

## Main

### Endocytosis Mechanisms and the Role of Membrane Tension

#### Endocytosis, Macropinocytosis, Phagocytosis

Endocytosis, the fundamental process of nutrient uptake and receptor signaling regulation, is utilized by virtually all nucleated cells of the body. The best described is clathrin-mediated endocytosis (CME) that proceeds through invaginations of the plasma membrane (PM) that sequester receptor-ligand complexes into ~50–200 nm pits coated with the protein clathrin (Figure 1) (Ehrlich et al., 2004; McMahon and Boucrot, 2011). These pits ultimately scission with the aid of the GTPase dynamin that constricts the neck of the bud, to form endocytic vesicles bearing cargo such as low-density lipoprotein (LDL), transferrin (Tf), and epidermal growth factor (EGF) bound to their cognate receptors (Kaksonen and Roux, 2018). Thus, while nutrients like lipids and iron are internalized for cell growth, CME also controls receptor signaling by removing receptors from the plasma membrane when needed (Tsao et al., 2001; Goh and Sorkin, 2013). Additionally, numerous clathrin-independent endocytic routes have been described including caveolae, tubulovesicular clathrin-independent carriers (CLICs), and fast endophilin-mediated endocytosis (FEME) (Mayor and Pagano, 2007). These pathways rely on specific biophysical properties for cargo internalization such as distinct membrane lipid compositions (i.e., lipid rafts), the engagement of the actin cytoskeleton to produce tubular invaginations or non-clathrin membrane shaping proteins (Galbiati et al., 2001; Doherty and McMahon, 2009; Boucrot et al., 2015).

In addition to one or multiple of these pathways, specialized cells also utilize macropinocytosis or phagocytosis to internalize large amounts of extracellular fluid or particulates, respectively. Macropinocytosis involves the rapid polymerization of cortical actin to produce plasma membrane ruffles that capture surrounding fluid and collapse to form large (>0.2 μm) internal vacuoles called macropinosomes (Swanson, 2008). Because this process results in the non-discriminant sampling of large amounts of extracellular fluid (Steinman et al., 1976), macropinocytosis is typically utilized by cells of the innate immune system to survey tissues for infection (West et al., 2004), as well as by some cancer cells as a means of sustaining the high nutrient requirements of their altered metabolic states (Commisso et al., 2013). In addition, professional phagocytes like macrophages are tasked with the removal of cell corpses and microbes, and as such utilize phagocytosis, a specialized form of endocytosis that allows for ingestion and degradation of particulates (Aderem and Underhill, 1999; Freeman and Grinstein, 2014).

#### Membrane Tension and Control of Endocytic Trafficking

It has been known for some time that PM tension regulates endocytic pathways (Gottlieb et al., 1993; Dai and Sheetz, 1995; Bajno et al., 2000). Low membrane tension is permissive of and induces multiple forms of endocytosis (Watanabe et al., 2013; Hirama et al., 2017; Wu et al., 2017; Loh et al., 2019). On the other hand, increases in tension oppose CME (Bucher et al., 2018) and require additional forces exerted by the actin cytoskeleton to complete pit formation and aid scission (Boulant et al., 2011). Yeast that have to invaginate their PM against turgor pressure are entirely dependent on their actin cytoskeleton for endocytosis (Aghamohammadzadeh and Ayscough, 2009). An interesting concept, however, is that cells are able to sense changes in membrane tension and exert control over it in order to govern membrane trafficking. Some of these control mechanisms have been described. For example, caveolae can assemble or disassemble to provide additional membrane as needed (Sinha et al., 2011; Golani et al., 2019), CLICs regulate membrane tension via the mechano-transducer vinculin (Thottacherry et al., 2018), and increases in PM tension during phagocytosis can signal to exocytosis machinery in order to deliver additional membrane as required (Masters et al., 2013). Thus, cells use endocytic machinery like caveolae and CLICs as well as exocytosis as important membrane reservoirs to provide rapid membrane slack when required. Conversely, cells must also have ways to prevent overt membrane slack, since acute decreases in membrane tension dysregulates endocytic uptake (Wu et al., 2017; Loh et al., 2019). Insights gained from studies on PM tension control raise questions regarding intracellular membranes: Do cells sense and regulate membrane tension throughout the endosomal-lysosomal system to direct endomembrane trafficking, and how? One way this could be achieved is by controlled osmotic shifts imposed by the transport of solutes. Numerous endolysosomal resident channels and transporters working in a concerted fashion may achieve such ends.

**Figure 2:**
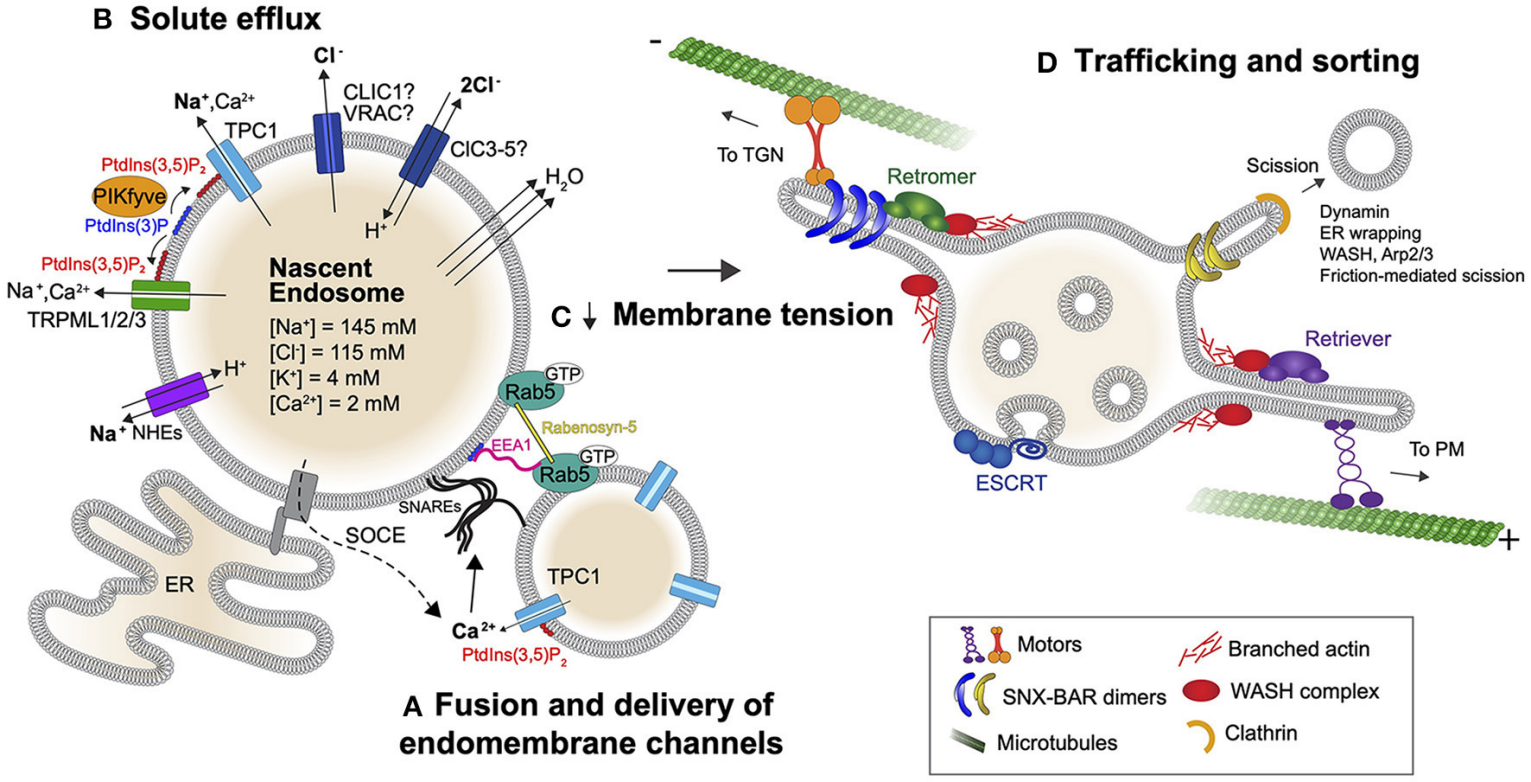
Lipid-gated monovalent ion efflux controls endomembrane tension and trafficking of early endosomes. **(A)** The fusion and delivery of endomembrane-resident channels including TPCs is important for the subsequent efflux of Na^+^ from the nascent endosome. Fusion is mediated by SNAREs in a Ca^2+^-dependent manner. The initial source of Ca^2+^ is not clear but can come from the endocytic fluid or via store operated calcium entry (SOCE). **(B)** Once incorporated into the endosome and activated by PtdIns(3,5)P_2_, TPCs and TRPMLs can transport Na^+^ down its concentration gradient into the cytosol. A parallel flux of Cl^−^ is also required, which could be facilitated by CLIC1, ClCs, or VRAC. At the near-neutral pH of early endosomes, ClCs allow for 2Cl^−^ to be transported out in exchange for 1H^+^. ClCs as well as Na^+^ proton exchangers (NHEs) may therefore contribute to early acidification of the endosome. Importantly, the efflux of Na^+^ and Cl^−^ from early endosomes drives osmotically obliged water out of the organelle and this fluid resolution reduces membrane tension. Rab5 may sense endomembrane tension as it drives endosome enlargement through effector proteins EEA1 and Rabenosyn-5 that facilitate SNARE-dependent fusion. **(C)** Fluid resolution ultimately lowers membrane tension. **(D)** Low membrane tension fosters the recruitment of membrane remodeling/sorting complexes to the endosome surface to drive early endosomal trafficking.

Immediately upon scission, endosomes containing cargo and fluid undergo rapid recycling of membrane. It should be obvious however, that without a parallel loss of fluid, membrane recycling would abruptly halt as hydrostatic pressure within the organelle would quickly build up to a point where membrane deformation is negated. A critical component of fluid resolution is that it accompanies efflux of osmolytes. While the composition of the extracellular fluid varies depending on the niche, it typically consists of a mix of organic solutes, macromolecules and small inorganic ions. Many of the molecules are in the form of polymers (i.e., polysaccharides, proteins, polynucleotides etc.) and are of relatively low concentrations, thus have a low osmotic contribution. There are some exceptions (including hydrating glucosaminoglycans) but these too are often of low abundance in most tissues. On the other hand, the types and concentrations of inorganic monovalent ions in extracellular fluids are high and kept relatively constant in the body; Na^+^ (140 mM) and Cl^−^ (110 mM) account for the major osmolytes present (Figure 2). Given that extracellular concentrations of Na^+^ and Cl^−^ are 3–10 times higher than the cytosol, this gradient favors the efflux of Na^+^ and Cl^−^ from nascent endosomes into the cytosol by channels. Indeed, recent work demonstrates that cells exploit this gradient to drive water out of vacuoles without costing the cell energy to relieve endomembrane tension and enable trafficking from the compartment (Freeman et al., 2020).

### Fluid Resolution Lowers Membrane Tension to Enable Early Endocytic Trafficking

#### Gated Efflux of Na^+^ Drives Endomembrane Remodeling and Trafficking

While all forms of endocytosis involve the uptake of fluid (pinocytosis), this is emphasized and can be readily monitored in cell types that *macro*pinocytose. In such cells, the newly formed macropinosomes resolve rapidly (within minutes) by way of shrinkage and extensive tubulation (Racoosin and Swanson, 1993; Steinman and Swanson, 1995). The compartments can also be manipulated by simply performing ion substitutions at the time of their formation and sealing. Monitoring the large endosomes when Na^+^ is substituted with non-transportable cations (e.g., N-methyl-D-glucosamine^+^), demonstrates that a Na^+^ gradient is necessary for tubulation and shrinkage of macropinosomes (Freeman et al., 2020). As a result, without Na^+^ the endosomes are large and perfectly spherical, as though swollen by trapped water. By inhibiting a panel of endolysosomal channels and transporters pharmacologically, Freeman et al. identified the Na^+^-conducting two-pore channels 1 and 2 (Wang et al., 2012; She et al., 2018) (TPC1 and TPC2) as key players in this process: Treatment of cells with the TPC inhibitor tetrandrine resulted in swollen macropinosomes. The resultant, distended macropinosomes, precluded the formation of membrane tubules that are a hallmark of early cargo sorting. Use of single and double knock-out strategies further revealed that TPC1 is primarily responsible for the resolution of volume from macropinosomes (Freeman et al., 2020) (Figure 2). Consistent with this finding, TPC1 has been shown to be recruited to macropinosomes immediately upon their formation, while TPC2 is recruited at later stages and operates at lysosomes (Calcraft et al., 2009; Freeman et al., 2020). Not surprisingly, macropinocytic cells like macrophages that handle large amounts of fluid experience pressure for Na^+^ efflux, as evidenced by their high expression of TPC1 (Freeman and Grinstein, 2018; Freeman et al., 2020). Nevertheless, TPC1^−/−^;TPC2^−/−^ mice are, for the most part, normal unless pressured to perform high membrane traffic (Grimm et al., 2014; Sakurai et al., 2015; Castonguay et al., 2017), suggesting either redundancy or compensation in Na^+^ efflux pathways in these animals.

The failure of macropinosomes to shrink and resolve without a Na^+^ gradient is due to the disruption of an efflux that supports the extrusion of osmotically coupled water from the vacuole (Figure 2). This loss of water is a critical trafficking prerequisite that lowers endomembrane tension by reducing the hydrostatic pressure within the vacuole, in turn rendering the vacuolar membrane amenable to deformation by trafficking complexes. Indeed, not only is macropinosome resolution impaired when a Na^+^ gradient is lost, but canonical receptor recycling pathways are disrupted in fibroblasts and epithelia cell types (Freeman et al., 2020). This suggests that the exploitation of a Na^+^ gradient is a universal mechanism used by cells to drive endomembrane trafficking. In principle, sodium proton exchangers (NHEs) could also efflux Na^+^ while causing early acidification of vacuoles (Nakamura et al., 2005), a process that would be electroneutral. Thus, while there are likely multiple pathways for vacuolar volume resolution, TPC1-mediated Na^+^ efflux represents a prototypical mechanism for relieving hydrostatic pressure and membrane tension to enable trafficking.

#### Counterion Flux Is Required for Volume Resolution

The efflux of Na^+^ by TPCs presents the need for counterion fluxes in order to maintain electroneutrality. It stands to reason that a parallel loss of Cl^−^ would be similarly necessary for the resolution of nascent endosomes. Indeed, ion substitution experiments in which Cl^−^ is replaced with the impermeant anion gluconate^−^ prevents macropinosome shrinkage and tubulation and the organelles again appear swollen, suggesting that water is trapped in the compartment (Freeman et al., 2020). The transport pathway for Cl^−^ is not known, but endolysosomal organelles bear several Cl^−^ channels and transporters that could potentially fulfill this role even in the early endosome. To this end, the Cl^−^ intracellular channel protein 1 may be involved in these counterion fluxes, however, its ion transport activities and mechanisms of action remain controversial (Stauber and Jentsch, 2013). Other endomembrane Cl^−^ transporters include members 3–7 of the ClC family of H^+^/Cl^−^ exchange transporters. Some of these transporters are located in early endosomes and can support early acidification by exchanging luminal Cl^−^ for H^+^ (Stauber and Jentsch, 2013), so like organellar NHEs, these could contribute to volume loss. Interestingly, gain of function mutations in members of the ClC family result in volume *increases* in the endocytic pathway (Nicoli et al., 2019), and ClCs are generally outwardly rectifying, so while these transporters have yet to show a role in the rapid process of volume resolution, they may contribute to the osmoregulation of endolysosomes in other ways. In addition, a recent screen identified LRRC8A, a component of the volume-regulated anion channel (VRAC), as necessary for the control of vacuolar volume, since inactivation of the *LRRC8A* gene causes swelling of endolysosomes (Lenk et al., 2019). Whether this effect is directly caused by the loss of VRAC activity from endosomes remains to be formally tested. Should VRAC function along the endocytic pathway (Li et al., 2020), its flux of Cl^−^ and that of several organic anions (Jentsch, 2016; Kasuya et al., 2018), could yield pleiotropic control over trafficking.

#### The Role of TRPMLs in Early Endomembrane Trafficking

Among the channels reported to play critical roles in membrane trafficking are the Ca^2+^ conducting transient receptor potential cation channels of the mucolipin subfamily 1-3 (TRPML1-3). Loss of TRPML1 causes severe vacuolation of endolysosomes in restricted cell types including gut epithelial cells (Venugopal et al., 2007) where only the expression of a wild type channel, but not pore-mutants, rescue this phenotype (Dong et al., 2010). TRPML1 is indeed rapidly acquired by nascent endosomes in myeloid cells but TRPML1^−/−^ macrophages do not appear vacuolated or impaired (SF unpublished). It should be noted that Ca^2+^ itself is a minor osmoticant of extracellular and endocytic fluid, with concentrations that change drastically in the endocytic pathway, but never reach >2 mM. The flux of Ca^2+^ from early endosomes may nevertheless be critical for early compartments to fuse with later ones via SNARE-mediated membrane fusion, analogous to proposed methods of secretory vesicle fusion with the plasma membrane (Park and Ryu, 2018). This would ensure that the endomembrane becomes endowed with the necessary channels and transporters that support fluid resolution (Figure 2). However, Ca^2+^ in the endocytic fluid is not ostensibly required for the shrinkage of nascent vacuoles (Freeman et al., 2020). This would suggest alternate sources of Ca^2+^ that contribute to fusion beyond that of the early endocytic fluid, potentially from previously formed endosomes. Despite being a minor osmotic contributor, Ca^2+^ indeed exits from early endosomes as its concentration quickly drops from 1 mM to low μM ranges despite volume loss (Scott and Gruenberg, 2011). The efflux of Ca^2+^ may be mediated by TRPML, TPCs, or even store-operated channels stimulated by contacts between the endoplasmic reticulum (ER) and the plasma membrane.

It remains unclear why TRPML1-deficient epithelial cells have enlarged endolysosomes. This could be either because (1) the compartment does not fuse to acquire TPCs or (2) because TRPMLs may be the critical mode of Na^+^ efflux in these cells. The former possibility stems from studies demonstrating that endosomal Ca^2+^, released by TRPML, regulates the fusion of endomembranes (Pryor et al., 2000; Dayam et al., 2015). Since TPCs exist in endomembrane reservoirs and are delivered to newly formed endosomes by fusion (Castonguay et al., 2017), their efficient delivery may require TRPML-mediated Ca^2+^ efflux. The latter is a possibility because TRPMLs are in fact non-selective cation channels, and were shown to be permeable to Na^+^ in addition to Ca^2+^ (LaPlante et al., 2002). Thus, TRPMLs may contribute to volume resolution either indirectly by aiding TPC delivery to endosomes or directly by mediating Na^+^ efflux along with TPCs. Either way, TRPML activity is necessary for the resolution of vacuolar fluid in select cell types. Interestingly, like some TRP channels in the PM, the gating of TRPML2 is mechanosensitive and activated under hypotonic (high tension) conditions (Chen et al., 2020). This is a remarkable finding, suggesting that channels that support volume loss are activated when biophysical demands present themselves.

#### A Critical Role for Phosphoinositides in Endomembrane Tension and Trafficking

TPCs and TRPMLs in fact belong to a class of ion channels that are gated by a single, rare lipid species found on the cytoplasmic leaflet of endomembranes—the phosphoinositide PtdIns(3,5)P_2_ (Dong et al., 2010; Wang et al., 2012; She et al., 2018). PtdIns(3,5)P_2_ is generated by a single kinase (PIKfyve) which is activated, in part, by osmotic stress (Gary et al., 1998; Bonangelino et al., 2002). This feature then confers the cell with an ability to control Na^+^ efflux in space, time and upon changes to ionic strength. Together with some understanding for how monovalent ion effluxes control volume resolution, the connections provide a new perspective on numerous studies reporting endolysosomal defects caused by the loss of PtdIns(3,5)P_2_. In yeast, it had long been reported that the loss of Fab1, the lipid kinase that synthesizes PtdIns(3,5)P_2_ by phosphorylating PtdIns(3)P, leads to the formation of abnormally enlarged vacuoles (Yamamoto et al., 1995; Gary et al., 1998). Subsequent studies in mammalian systems further demonstrated extensive vacuolation of endolysosomal compartments upon PtdIns(3,5)P_2_ depletion (Ikonomov et al., 2001; Rutherford et al., 2006; Chow et al., 2007; Zhang et al., 2007; Jefferies et al., 2008; Zolov et al., 2012; Cai et al., 2013; Sharma et al., 2019). That PtdIns(3,5)P_2_-deficient vacuoles are large, phase-lucent, highly spherical organelles, suggests that they are fluid-filled and likely experience high membrane tension that precludes the formation of tubules.

Supporting this notion, loss of PtdIns(3,5)P_2_ blocks endosome fission (Sharma et al., 2019), prevents receptor recycling (Freeman et al., 2020) and trafficking of the proton-pumping vacuolar-type ATPase (V-ATPase) (Buckley et al., 2019), impairs retrograde transport of numerous cargoes to the TGN (Rutherford et al., 2006), and arrests phagosome resolution (Krishna et al., 2016). Importantly, all of these processes proceed through membrane deformation events such as budding, tubulation, and scission. In addition, the acute washout of inhibitors of PIKfyve, promptly results in extensive tubulation, vesiculation, and shrinkage of the engorged endolysosomes (Sharma et al., 2019; Freeman et al., 2020). Distended macropinosomes in PIKfyve-inhibited cells can be induced to shrink and form tubules when subjected to hypertonic solution (Freeman et al., 2020), a condition that osmotically forces water out the cell and concomitantly, out of the vacuoles. These findings suggest that PtdIns(3,5)P_2_ controls the vacuolar efflux of Na^+^ through its gating of TPCs and TRPMLs, and that this in turn drives water out of the lumen and triggers membrane remodeling processes by reducing membrane tension. Like all phosphoinositides, the effectors of PtdIns(3,5)P_2_ collectively orchestrate a gamut of cellular functions. For example, in yeast, this phosphoinositide can regulate the assembly of the V-ATPase (Li et al., 2014) yet lysosome swelling is prevented when mutating or inhibiting the pump in PtdIns(3,5)P_2_-deficient cells (Wilson et al., 2018; Sharma et al., 2019). It seems likely, therefore, that PtdIns(3,5)P_2_ may support efflux of Na^+^ and Ca^2+^ but also govern ion and specifically proton transport at the lysosome in yet poorly-defined ways.

#### Endomembrane Tension Sensing

A major outstanding question in the field is how endosomes sense their luminal contents to direct traffic. As previously alluded to, cation channels (e.g., TRPML), PIKfyve, and VRAC/LRRC8 are all responsive to endomembrane tension and/or osmotic stress, suggesting that there are numerous feedback mechanisms for the cell to calibrate ion transport to the tension experienced at organelles (Bonangelino et al., 2002; Chen et al., 2020; Li et al., 2020). Tension on the endomembrane may impact lipid packing or spacing of lipid headgroups and could conceivably recruit or activate signaling complexes that sense these events. In this regard, given that the small GTPases of the Rab family control nearly all aspects of membrane traffic, including vesicle budding, docking, fusion, and transport (Grosshans et al., 2006), they would seem to be likely candidates. Like all GTPases, Rabs function as molecular switches; when GTP-bound they recruit effector proteins with diverse functions in membrane trafficking, whereas they are inactivated by GTPase activating proteins (GAPs) that help convert the GTP to GDP. The cycle repeats when the GDP is removed by a guanine nucleotide-exchange factor (GEF) and the GTPase is loaded with GTP once again. This ability to rapidly cycle between on and off states and their individual specificities for membrane compartments makes Rab proteins well-suited for the task of sensing membrane tension to direct traffic. Rab5 in particular is a possible candidate in as much as its activation can disrupt volume and trafficking of early endosomes. Enlarged endosomes are observed with a constitutively activated mutant of Rab5 (Stenmark et al., 1994; Roberts et al., 2000; Murray et al., 2002; Galperin and Sorkin, 2003), as well as upon ectopic expression of its GEF (Otomo et al., 2003) or mutation of its GAP (Sun et al., 2012). This phenotype is accompanied by impaired transferrin recycling and the retention of transferrin receptor in enlarged endosomes (Stenmark et al., 1994; Sun et al., 2012).

The endosome enlargement under Rab5 activating conditions is attributed to the recruitment of several Rab5 effectors that facilitate SNARE-dependent membrane fusion, including EEA1 and Rabenosyn-5 (Simonsen et al., 1998; Christoforidis et al., 1999; Nielsen et al., 2000). Moreover, Rab5 is required for the endosomal enlargement in PIKfyve inhibited cells (Compton et al., 2016). When considering the extreme size of PtdIns(3,5)P_2_-deficient vacuoles, and of those produced by inhibition of TPCs or Na^+^ removal, it is clear that while deformation of the turgid membrane is hindered, incoming membrane fusion with this compartment is not impaired. This raises the possibility that Rab5 senses membrane tension to induce fusion, which may be a means to offset some of the tension by providing additional membrane to this stressed system.

#### Endomembrane Remodeling and Trafficking as a Consequence of Membrane Tension Relief

Upon internalization, various receptors and their ligands are simultaneously sorted into membrane subdomains of the early endosome and trafficked to specific destinations. Transferrin and its receptor are recycled to the plasma membrane (Dautry-Varsat et al., 1983; Dunn et al., 1989) in order to bind and take up more iron (Klausner et al., 1983), acid-hydrolase receptors are retrieved back to the TGN after delivering newly synthesized hydrolases to endosomes (Bonifacino and Rojas, 2006), and cargo such as LDL and EGF bound to its receptor are delivered to lysosomes and degraded (Carpenter and Cohen, 1979; Brown et al., 1983; Dunn et al., 1989). The endomembrane subdomains within which these sorting events occur are morphologically distinct (Mellman, 1996). For example, recycling cargo enters slender membrane tubules that pinch off to form tubular and vesicular transport carriers while remaining cargo is sequestered into inward-budding membrane invaginations that form intralumenal vesicles (ILV) within the endosome. The former, in fact, has also been described as a tubular endosomal network in which multiple interconnected membrane tubules are formed from an endosomal subdomain (Bonifacino and Rojas, 2006).

The formation of tubular subdomains on endosomes is orchestrated by the recruitment and assembly of trafficking complexes, such as retromer that targets cargo to the TGN (Figure 2) (Seaman et al., 1998). Retromer is composed of a heterotrimeric cargo selective complex that recognizes cargo in endosomal membranes, as well as a heterodimer of SNX-BAR proteins (combinations of SNX1, SNX2, SNX5, SNX6) that bind and stabilize highly curved membrane tubules (Carlton et al., 2004; Wassmer et al., 2007). Interestingly, the SNX-BAR proteins can also function independently of retromer in cargo trafficking (Kvainickas et al., 2017; Simonetti et al., 2017). SNX5/6 heterodimers and the SNX-BAR protein SNX4 can additionally interact with components of the retrograde microtubule motor protein dynein (Traer et al., 2007; Wassmer et al., 2009) to direct trafficking from endosomes to a perinuclear endosomal recycling compartment (Traer et al., 2007). In addition, endosomal tubules decorated with other SNX-BARs were shown to be affected by perturbations in dynein or the anterograde microtubule motor kinesin (Hunt et al., 2013). Membrane tubule interactions with motors likely contributes to their elongation and scission (Hunt et al., 2013), by their stretching along microtubules.

Retromer can also associate with WASH, which regulates branched actin polymerization on the endosome to facilitate membrane remodeling, cargo sorting and membrane scission (Derivery et al., 2009; Puthenveedu et al., 2010; Buckley et al., 2016). Furthermore, the recently identified complex, retriever, that shares striking similarity to retromer, also interacts with WASH on endomembranes and directs cargo trafficking toward the plasma membrane (McNally et al., 2017). The membrane tubules that form can be further remodeled into buds, by the recruitment of the coat protein clathrin (Stoorvogel et al., 1996), that eventually scission into smaller cargo-carrying vesicles (Saint-Pol et al., 2004). Membrane scission at these sites was shown to be mediated by the collar-forming GTPase dynamin (Llorente et al., 1998; Nicoziani et al., 2000), contact sites with the endoplasmic reticulum (Rowland et al., 2014) and through friction mediated scission (Simunovic et al., 2017).

Remnant cargo that escapes sorting into tubules may be directed into ILVs as the endosome matures into a multivesicular body. ILVs are formed by the endosomal sorting complex required for trafficking (ESCRT), that assembles as spiral filaments on the surface of endomembranes to drive inward membrane budding and constriction (Figure 2) (Pfitzner et al., 2020). ESCRT is made up of 4 subcomplexes: ESCRT-0 mediates initial binding to endomembranes and recruits ESCRT-I, which in turn recruits ESCRT-II, followed by ESCRT-III, the key functional subcomplex that drives membrane deformation and scission along with the ATPase VPS4 (Saksena et al., 2009; Adell et al., 2014; Chiaruttini et al., 2015; Maity et al., 2019). The inward budding events generated by ESCRT-III result in the delivery of membrane receptors into the lumen of the organelle, where they are degraded by subsequent fusion with lysosomes (Katzmann et al., 2002).

All of these membrane remodeling events including tubulation, scission, and invagination that are sculpted by coat proteins and the spiral polymerization of ESCRT involve extreme deformations to the endomembrane. Modeling predictions and solved structures for BAR domain-containing SNXs show preferred binding to tubules of 20–60 nm in diameter (Mim et al., 2012; Simunovic et al., 2015) which is supported by reconstitution experiments (see van Weering et al., 2012 for example). ESCRT-driven invaginations can be even more narrow (Pfitzner et al., 2020). In the case of tubules, the substructures can reach remarkable lengths, even several microns (Kerr et al., 2006). Given the substantive changes in the surface to volume ratios, it follows that to create these features, membrane-deforming proteins require low membrane tension for their assembly while high hydrostatic pressure increases membrane tension which offsets the process altogether (Zimmerberg and Kozlov, 2006; Shi and Baumgart, 2015; Simunovic and Voth, 2015). This notion was empirically tested by exposing liposomes to hypertonic solutions which leads to their crenation and the recruitment of the BAR domain-containing protein BIN1, which in turn induces liposome tubulation. Conversely, swelling the liposomes prevents the BIN1-mediated tubulation (Freeman et al., 2020). And, as expected, ESCRT-III-mediated ILV formation has a similar dependency on low membrane tension (Booth et al., 2019; Mercier et al., 2020) and prefers assembly with curved/crenated membranes (Lee et al., 2015). Thus, membrane tension relief that is accomplished via the efflux of monovalent ions and the concomitant extrusion of water, is a critical event that maintains a low hydrostatic pressure and triggers membrane crenation. This crenation in turn lowers the energy barrier for membrane deformation by proteins like SNX-BAR and ESCRT-III that maintain membrane trafficking.

Failure to relieve endomembrane tension disrupts cargo trafficking and is incapacitating. Treatment of cells with PIKfyve inhibitors or other compounds that cause endosomal swelling impairs retrograde trafficking (Rutherford et al., 2006), recycling of cell surface receptors (Carpentier et al., 2013; Freeman et al., 2020) and leads to cell death (Overmeyer et al., 2011; Martin et al., 2013). In addition, knock-out of TPC1 or inhibition of PIKfyve or TPCs, causes decreased responsiveness of the tumor cell line HT1080 to EGF, and results in delayed growth (Freeman et al., 2020). Disruption of the Na^+^ gradient by culturing cells in Na^+^-free medium, prevents the recycling of Mac-1 (α_M_β_2_-integrin) from endosomes to the plasma membrane and impairs phagocytosis and formation of focal adhesions, processes that require surface integrins (Freeman et al., 2020). The importance of Na^+^ efflux was demonstrated *in vivo* as well. Laser ablation of tissue to mimic small injuries such as those that may occur during exercise, normally causes the migration of highly macropinocytic resident tissue macrophages to injury sites to contain the damage (Uderhardt et al., 2019). However, when vacuolar Na^+^ efflux is prevented, the macrophages fail to resolve their macropinososmes and are unable to survey their environment and respond to the damage. This results in neutrophil “swarming” of the affected area, an aberrant and inflammatory response (Freeman et al., 2020). Thus, cells exploit a Na^+^ gradient to resolve fluid from their endosomes and maintain the low membrane tension required for trafficking and overall cell responsiveness.

### Lysosomal Solute Efflux and Membrane Trafficking

Maturing endosomes and autophagosomes ultimately fuse with lysosomes in order to degrade their luminal contents. The fusion with lysosomes confers on these hybrid organelles degradative properties by delivering the V-ATPase to the limiting membrane and soluble acid hydrolases to the endocytic fluid. But (endo)lysosomes are not just terminal compartments where breakdown occurs. Instead, they function in membrane repair, immunity, and are major signaling hubs that posit at the crossroads of nutritional status, transcription/translation, and cellular homeostasis. To meet the demand of such diverse functions, lysosomes are remarkably dynamic organelles. While the steady-state distribution of lysosomes in the cytoplasm is typically perinuclear, with some occupying the peripheral cytosol, they undergo frequent bi-directional transport between these areas (Pu et al., 2016). Lysosomes also undergo fusion, fission and tubulation by coupling to the motor proteins dynein and kinesin (Phaire-Washington et al., 1980; Swanson et al., 1987; Luzio et al., 2007; Mrakovic et al., 2012; Saffi and Botelho, 2019). Indeed, these properties are implicated in many of their functions including encounter and fusion with autophagosomes (Jia et al., 2017), antigen presentation (Vyas et al., 2007; Garg et al., 2011; Saric et al., 2016), exocytosis (Rodriguez et al., 1997; Tuli et al., 2013; Encarnação et al., 2016), and their reformation following autophagy (Yu et al., 2010). Remarkably, and germane to the principles discussed in this review, at least the signaling pathways known to be initiated at the lysosomal membrane are engaged according to the luminal solutes of the lysosome including amino acids, protons, and we will argue, major osmoticants (Sancak et al., 2008; Zoncu et al., 2011).

**Figure 3:**
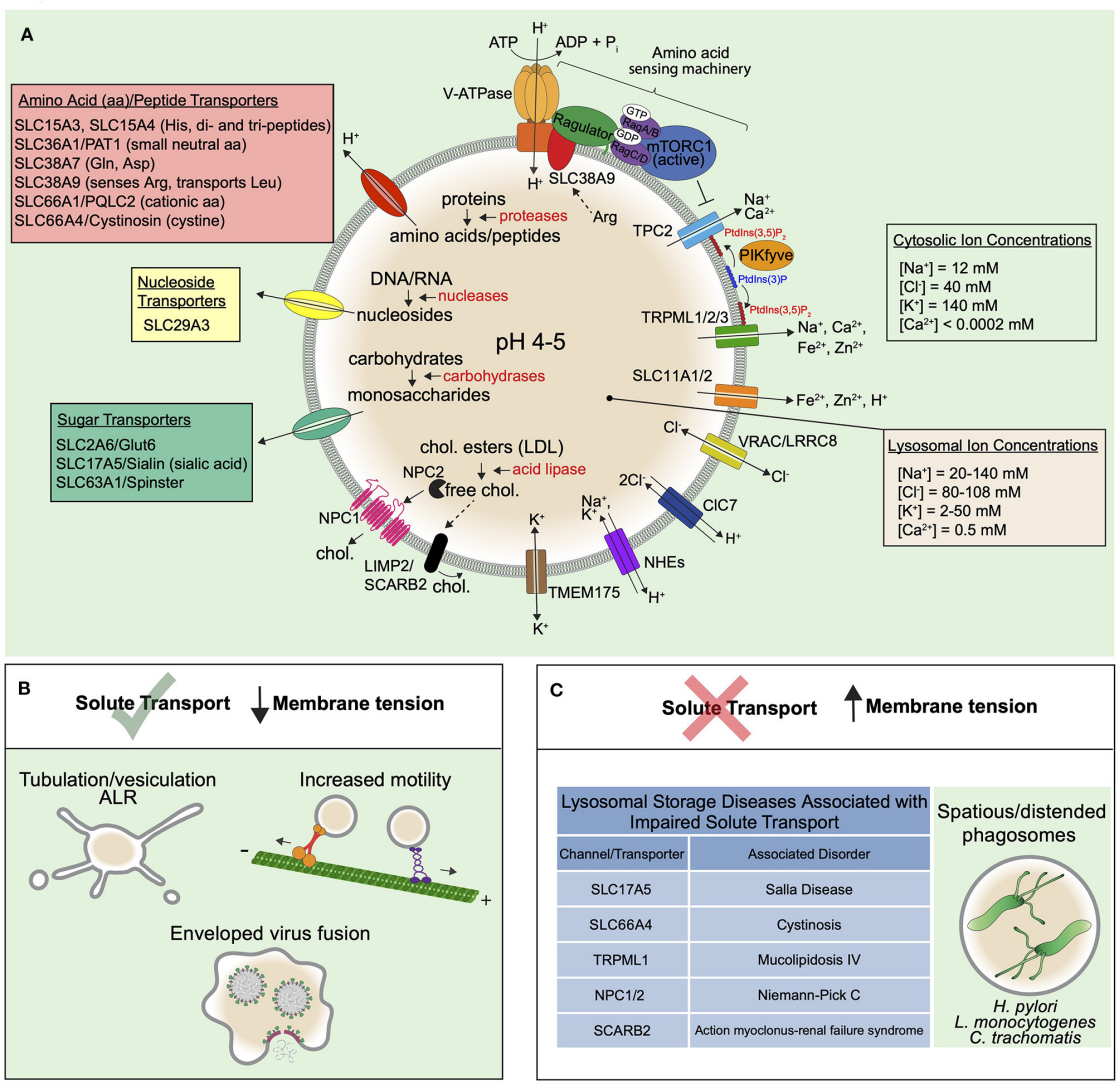
Solute transport controls the trafficking and function of lysosomes. **(A)** Low lysosomal pH (4-5) is maintained by the V-ATPase that uses energy from ATP hydrolysis to constitutively pump H^+^ into the lumen. The low pH is supported by a counter-ion flux involving either the efflux of cations or influx of anions. The luminal Na^+^ and Cl^−^ concentrations of the lysosome are higher than that of endosomes, suggesting that both mechanisms may be involved. Cl^−^ transport by ClC7 is indeed important for maintaining proper lysosomal functions in certain cell types. VRAC/LRRC8 also features in Cl^−^ transport in lysosomes. Since lysosomal NHEs may exchange either Na^+^ *or* K^+^ for H^+^, the K^+^ channel TMEM175 may also be critical for the control of lysosome size, fission, and tension. Indeed, to remodel the limiting membrane of the lysosome, the extrusion of major osmolytes is thought to be a necessary prerequisite and may orchestrate the process altogether. In this regard, it is known that Na^+^ efflux, as activated by PtdIns(3,5)P_2_, causes the extrusion of water from lysosomes, leading to a lowering of the hydrostatic pressure and subsequent membrane tension. PtdIns(3,5)P_2_ also regulates the V-ATPase either directly or indirectly. The low pH of the lysosome maintains the activity of various acid hydrolases (red text) in the luminal fluid that break down the macromolecules that reach lysosomes by various endocytic uptake pathways and autophagy (see Figure 1). The building blocks generated from this hydrolysis are exported out of lysosomes via numerous transporters of the SLC family depicted and described in the main text. Known solutes for the SLC transporters are provided in parentheses next to transporter names. The best described example is the amino acid transporter SLC38A9 that senses luminal arginine to activate mTOR on the lysosomal surface via complexes collectively termed the “amino acid sensing machinery.” mTOR also binds and inhibits TPCs, providing a link between amino acid sensing and membrane tension control. Cholesterol efflux is also well-described and is exported from lysosomes by NPC1/NPC2 proteins as well as SCARB2. In general, solute efflux is critical for nutrient acquisition but also to prevent an osmotic pressure that could increase lysosomal hydrostatic pressure and membrane tension. **(B)** When solute efflux via lysosomal channels and transporters is functioning optimally, lysosomal membrane tension is controlled and this supports trafficking processes like ALR, that proceeds through lysosome tubulation, lysosomal motor-driven motility, and viral fusion. **(C)** When solute efflux mechanisms are impaired, this leads to lysosomal storage disorders, many of which exhibit distended lysosomes and likely high membrane tension that precludes lysosome tubulation and trafficking. In addition, some bacteria may modulate lysosomal membrane tension for their benefit.

Importantly, because multiple pathways converge on lysosomes, this relatively small organelle that normally occupies a fraction of the total cell volume (~2.5% Steinman et al., 1976) experiences high solute flux. As such, the lysosome is equipped with a suite of channels and transporters that maintain its ionic composition and facilitate the flux of catabolites from the lumen (Figure 3). Maintenance of its ionic composition is critical for proper acidification, membrane potential, and in order to remain iso-osmotic with the cytosol (Xu and Ren, 2015). The luminal concentrations of mono- and di-valent inorganic ions have been estimated or determined with ratiometric dyes or by isolating/patch-clamping lysosomes, though values have a wide range owed to challenges with each of these approaches. Na^+^ concentrations have been determined as between 20 mM and upwards of 140 mM for example (Steinberg et al., 2010; Morgan et al., 2011; Wang et al., 2012), though many experimental approaches suggest it is the major cation of the lysosome (Morgan et al., 2011). Measurements of lysosomal Cl^−^ come with similar challenges but largely show a tighter spectrum of estimates ranging from 60 to 110 mM (Sonawane and Verkman, 2003; Morgan et al., 2011; Stauber and Jentsch, 2013; Saha et al., 2015) suggesting Cl^−^ is the major anion of the compartment. Although it is not known if or how much osmotic pressure lysosomes may experience, dysregulation in Na^+^ or Cl^−^ transport are expected to cause osmotic imbalances and high membrane tension and we describe this in the following subsections. Moreover, well-characterized storage disorders of the lysosome that cause the accumulation of organic osmolytes will also drive such hydrostatic pressure and membrane tension to build up at the lysosome. We therefore also discuss organic solutes and broadly describe osmoregulation of lysosomes in homeostasis and disease.

#### The V-ATPase and Counterion Fluxes in Endolysosome pH: Osmotic Considerations

Maintaining a low lysosomal pH (4.5-5) aids in enzymatic digestion, is important for lysosomal trafficking, and the steep proton gradient supports lysosomal cotransporters whose activities are H^+^-coupled. The low pH is maintained by the V-ATPase, a pump that consumes ATP to constitutively transport protons into the organelle (Figure 3). However, this process is electrogenic and the highly positive charge generated within the lumen would quickly inhibit further proton pumping. To circumvent this problem, an ion counter-flux exists to dissipate the electrical potential across the membrane (Fuchs et al., 1989). Theoretically, this can be done either by adding negative charge or removing positive charge from the lumen. From an osmotic standpoint, the addition of negative charge leads to an osmotic gain in the compartment while removal of positive charge does not.

Despite the osmotic consequences, the addition of negative charge is not just theoretical; it is known to be accomplished by Cl^−^ transporters including the ClC7 H^+^/Cl^−^ exchange transporter that brings 2Cl^−^ in for 1H^+^ out (Stauber and Jentsch, 2013) such that the V-ATPase can bring 3H^+^ in. In the absence of ClC7, osteoclasts cannot remodel bone and mice and patients become severely osteopetrotic (Kornak et al., 2001). Moreover, the loss of ClC7 causes neurodegeneration and lysosomal storage disease (Kasper et al., 2005), so clearly ClC7 is important in pH regulation. It is curious therefore that numerous studies have demonstrated that ClC7 is in fact not required for lysosomes to reach their acidic pH (Kasper et al., 2005; Weinert et al., 2010). The discrepancies between the observed physiological phenotypes and that of single lysosomes in their steady state needs to be rectified. It should be noted, however, that certain circumstances require rapid and deep transitions in the pH of compartments, yet these events are rarely captured experimentally. Examples include host-pathogen interactions where a phagosome has little time to waste in acidifying to become a hostile environment for the microbe. This could equally be the case for the osteoclast lacunae—a large compartment with a seal that evolves along with the substratum being degraded. In the case of the osteoclast lacunae, the extracellular osmotic gain of Cl^−^ is of no consequence, so using Cl^−^ efflux to drive the V-ATPase would be advantageous in this setting. For the maturing phagosome, perhaps the tradeoff between contending with a little volume gain and making a steep transition in pH is one the cell is willing to make. Either way, given the osmotic consequence of Cl^−^ transport, control over ClC7 activity is critical. Indeed, gain-of-function mutations in ClC7 cause massive swelling of lysosomes (Nicoli et al., 2019).

Once a low pH is reached, the steady state is supported by ongoing activity of the V-ATPase coupled to a proton “leak” as mediated by solute transport systems (see below) and alkali cation exchangers (Casey et al., 2010). The latter necessitates the efflux of cations to prevent their accumulation and support electro- and osmo-neutrality (Steinberg et al., 2010). Since lysosomal [Na^+^] has been estimated to be high, it is interesting to speculate on the role of TPC2 and Na^+^ transport from the lysosome. TPC2 may play a similar role at the lysosome as TPC1 at the endosome—to move Na^+^ down its concentration gradient (from the lumen to the cytosol), in this case to promote acidification as well as prevent osmotic swelling that opposes trafficking. In this regard, lysosomes are indeed more alkaline in skeletal muscle cells of TPC2^−/−^ mice compared to wild type animals (Lin et al., 2015). And, interestingly, overexpression of TPC2 alone in HeLa cells causes extensive lysosome tubulation and increases lysosomal motility, whereas overexpression of a pore-mutant prevents tubulation (Freeman et al., 2020). It cannot be disregarded that proton exchangers of lysosomes may equally transport K^+^ (Wilson et al., 2018), which would also require a K^+^ channel that drives its efflux. This could involve TMEM175, a bidirectional K^+^ channel that is essential to maintain normal lysosome biology, especially in neurons (Cang et al., 2015).

While little is known about the role of Na^+^ in the lysosome, Ca^2+^ is much better appreciated. Amongst its many functions, Ca^2+^ is an important second messenger in signal transduction pathways and regulates membrane fusion (Clapham, 2007), thus, it's release from lysosomes must be transient and exquisitely timed. This is nicely reviewed elsewhere (Morgan et al., 2011). From an osmotic view, although the concentration of Ca^2+^ in the lysosome is much higher than that of the cytosol ([Ca^2+^]_lysosome_ = 0.5 mM (Christensen et al., 2002), [Ca^2+^]_cytosol_ <0.0002 mM), it is of low abundance compared to Na^+^ and K^+^, and Ca^2+^ efflux also does not appear to play a role in the acidification of lysosomes (Steinberg et al., 2010). Still, the timed release of Ca^2+^ from lysosomes is important for fusion events as mediated by TRPML (Pryor et al., 2000; Dayam et al., 2015). Similar to endosomes, lysosomal TRPML channels and PtdIns(3,5)P_2_ likely contribute to the lipid-gated control of Ca^2+^ efflux to support trafficking.

#### Controlled Lysosomal Solute Efflux Governs Membrane Tension and Trafficking

Maintaining lysosomal acidity is critical for the luminal hydrolases, including proteases like most cathepsins, various carbohydrate-processing enzymes, and nucleases and lipases that function optimally at low pH. Collectively, these enzymes digest macromolecules into their constituent building blocks. These solutes are in turn effluxed from lysosomes via solute transporters, in order to be used by the cell in anabolic reactions that sustain growth. An additional consideration is that in the absence of such efflux, the osmotic burden from enzymatic hydrolysis would be substantial. For example, a single internalized polysaccharide of 100 glucose monomers, when enzymatically processed, generates 100 osmotically active monosaccharides. If solute efflux mechanisms did not exist, the resulting inward flow of water due to a 100-fold higher osmotic gradient would generate hydrostatic pressure against the compartment. Thus, cells continually efflux lysosomal solutes to prevent osmotic swelling and overtly high membrane tension, which in turn supports lysosomal resolution by way of tubulation, vesiculation, and trafficking. Cells likely utilize these pathways to orchestrate endomembrane remodeling events that are coincident with organellar resolution and fission.

A clue to this comes from studies on autophagic lysosome reformation (ALR): Lysosomes that fuse with autophagosomes to form autolysosomes, reform by way of tubulation and fission only upon completion of autophagy (digestion) (Yu et al., 2010). The molecular machinery that facilitates lysosomal membrane remodeling remains to be discovered, however, some coat proteins have been described. For example, the adaptor protein complex AP5 may function with the hereditary spastic paraplegia proteins SPG11 and SPG15 as a novel coat-like complex to retrieve mannose 6-phosphate receptors from late endosomes/lysosomes to the TGN (Hirst et al., 2013, 2018). Consistent with this, enlarged lysosomes are observed in SPG11- or SPG15-depleted cells (Renvoise et al., 2014), and these proteins have been shown to mediate ALR (Chang et al., 2014). Also, clathrin was shown to mediate ALR tubule formation (Rong et al., 2012), suggesting that lysosome trafficking may be similarly dependent on membrane tension as endocytosis and early endosomal sorting. In this regard, it is remarkable that TPC2^−/−^ animals show defects in autophagic flux (Garcia-Rua et al., 2016). Putatively, Na^+^ efflux from the autolysosome orchestrates endomembrane remodeling and ALR but how this is timed remains unclear and the [Na^+^] of autophagosomes/autolysosomes has not been estimated to our knowledge. It is nevertheless enticing to anticipate that monovalent ion efflux must be choreographed such that organic solutes are fluxed before resolution occurs so as not to defeat the autophagic function.

#### The Role of the SLC Transporters in Lysosomal Solute Efflux and Membrane Tension Control

The lysosomal efflux of organic solutes is carried out by a suite of transporters belonging to the solute carrier (SLC) protein families (Figure 3). When solute efflux is prevented, osmotically-imposed lysosomal swelling occurs, a condition that limits lysosomal transport (Bandyopadhyay et al., 2014). An indication that solute efflux mitigates high membrane tension comes from investigation of sucrosomes. When sucrose, a disaccharide of glucose and fructose that is resistant to cleavage by lysosomal enzymes, is loaded into lysosomes by endocytosis, lysosomes swell (Cohn and Ehrenreich, 1969), suggesting that water is trapped in the compartment. The introduction of invertase, an enzyme that breaks down sucrose into its monosaccharide constituents, induces a prompt shrinkage and, interestingly, extensive tubulation of the lysosome (Swanson et al., 1986; Bright et al., 2016). Since glucose and fructose can be transported out of lysosomes by unknown monosaccharide transporters (Mancini et al., 1990; Lizak et al., 2019), it is likely that efflux of this sugar drives the osmotic extrusion of water from the organelle. Consistent with this, mutations in SLC17A5, a H^+^-driven acid sugar transporter, cause Salla disease, a rare lysosomal storage disorder (LSD) in which sialic acid is unable to be exported out of lysosomes (Aula et al., 1979; Renlund et al., 1986; Verheijen et al., 1999; Tarailo-Graovac et al., 2017). Consequently, vacuolated endolysosomes are observed in patient peripheral blood lymphocytes (Aula et al., 1979). In addition, loss of Spinster (Spin), another lysosomal sugar transporter, also causes lysosomal enlargement in cultured NRK cells. Remarkably, the loss of Spin alone blocks lysosome tubulation during ALR (Rong et al., 2011). The requirement for Spinster and other sugar efflux pathways would be especially pronounced in cells undergoing glycophagy or those internalizing polysaccharides from their surrounding fluid. As the efflux of monosaccharides is required to maintain the low membrane tension to support trafficking, cells under these conditions should be investigated with interest.

The efflux of amino acids is equally essential. Numerous lysosomal amino acid transporters have been identified including SLC66A4/Cystinosin, SLC36A1/PAT1, and SLC66A1/PQLC2 that transport cystine (Town et al., 1998), small neutral amino acids (Sagne et al., 2001) and cationic amino acids (Liu et al., 2012). Hematopoietic cells additionally express the proton-coupled transporters SLC15A3 and SLC15A4 that transport histidine as well as di- and tri-peptides. Their activities are particularly important in innate immune responses such as sensing of bacterial peptides from phagosomes (Nakamura et al., 2014) and mast cell functions (Kobayashi et al., 2017). Indeed, loss of some of these amino acid/oligopeptide transporters was also shown to cause lysosome enlargement (Liu et al., 2012; Kobayashi et al., 2017). In addition, the sodium-coupled neutral amino acid transporters SLC38A7 and SLC38A9 transport glutamate and asparagine (Hagglund et al., 2011; Verdon et al., 2017) and leucine among other amino acids (Wyant et al., 2017). The case of SLC38A9 is particularly intriguing as it not only transports amino acids, but is able to sense luminal arginine and relay this information to activate mTORC1 on the lysosome surface (Zoncu et al., 2011; Rebsamen et al., 2015; Wang et al., 2015). This relay occurs through physical interactions between several proteins and complexes spanning both sides of the lysosomal membrane, that collectively can be termed the “amino acid sensing machinery” (Figure 3). And, in addition to SLC38A9, PAT1 can also activate mTOR on lysosomes (Ogmundsdottir et al., 2012). Of note, mTOR interacts with and inhibits TPCs, while nutrient deprivation, a condition that inactivates mTOR and causes its dissociation from lysosomes, constitutively opens the channels (Cang et al., 2013). Given that TPCs can lower membrane tension via Na^+^ efflux, and that their activity supports tubulation and trafficking, perhaps mTOR activation via the amino acid sensing machinery simultaneously prevents lysosomal vesiculation and resolution, until sensing is complete. This raises the possibility that mTORC1 could contribute to membrane tension sensing.

Like sugars and amino acids, polynucleotides are another major type of biological polymer that is degraded within lysosomes. Sources of these can come from the DNA and RNA of dead cells that are continually being cleared by phagocytosis (Hochreiter-Hufford and Ravichandran, 2013). Following the breakdown of polynucleotides by lysosomal hydrolases such as acid deoxyribonuclease (Odaka and Mizuochi, 1999), the efflux of their monomers is necessary to prevent excessive osmotically imposed increases in lysosomal membrane tension. To this end, the lysosomal nucleoside/nucleobase transporter SLC29A3 (also known as ENT3), functions to efflux nucleosides in a pH-dependent manner (Rahman et al., 2017). Importantly, splenic macrophages from ENT3^−/−^ mice exhibit swollen lysosomes (Hsu et al., 2012), signifying an osmotic defect that likely precludes lysosome tubulation and trafficking.

Phagocytosed cell corpses (and autophagosomes) are also a significant source of membranes, which are largely composed of phospholipids, glycolipids and cholesterol. These lipids are broken down by various lysosomal enzymes (Schulze et al., 2009) into individual monosaccharides, long chain bases and fatty acids. These products also need to be exported out of lysosomes though the transport pathways involved are poorly described. The one exception is with cholesterol, a significant source of which comes from endocytosed LDL particles, where a single known lysosomal acid lipase converts the LDL-derived cholesteryl esters into free cholesterol. The free cholesterol is transferred to the transmembrane protein Niemann Pick C1 (NPC1) (Infante et al., 2008; Kwon et al., 2009; Li et al., 2016) by the luminal protein NPC2. Cholesterol may also be transported to the limiting membrane by lysosomal integral membrane protein 2 (LIMP2/SCARB2) (Heybrock et al., 2019). Once incorporated into the lysosomal membrane, cholesterol is exported out of lysosomes to reach various other cellular destinations by vesicular and non-vesicular means (Luo et al., 2019) (Figure 3). Lysosomes can make direct contacts with the endoplasmic reticulum (ER) to transfer cholesterol down a concentration gradient via transport protein complexes (Hoglinger et al., 2019). Mutations in NPC proteins lead to Niemann Pick type C disease, a severe lysosomal storage disorder characterized by massive cholesterol accumulation within lysosomes (Vanier, 2010). As a result, lysosomes are enlarged (Lim et al., 2019), and, although in this case the enlargement is not attributed to an osmotic effect, the membrane tension may be higher than normal. Interestingly, mTORC1 is constitutively activated in NPC1^−/−^ cells (Lim et al., 2019), a condition that also inhibits TPCs and Na^+^ efflux (Cang et al., 2013). Finally, it cannot be ruled out that the direct insertion of lipids into the lysosomal limiting membrane may itself affect membrane tension and lysosomal trafficking: Such a contribution is complex since cholesterol would increase total membrane surface area while also increasing membrane order and effecting lipid packing (Hofsab et al., 2003; Zimmerberg and Kozlov, 2006).

#### Lysosomal Storage Disorders Are Associated With Impaired Solute Efflux

The inability to transport solutes across the lysosomal membrane results in their accumulation in the lumen and is the cause of numerous lysosomal storage disorders (LSDs), a class of inherited metabolic diseases. At least 70 LSDs have now been identified and we refer the reader to several excellent reviews describing the different types (Futerman and van Meer, 2004; Platt et al., 2018). Majority of LSDs are caused by mutations in genes encoding soluble lysosomal enzymes. This typically leads to lack of or incomplete digestion of their substrates. As a result, transportable products of these macromolecules are not formed and the substrates accumulate in the lumen, as they themselves are unable to be exported from lysosomes. A smaller category of LSD are caused by mutations in genes encoding lysosomal channels and transporters (Figure 3). Interestingly, many LSDs exhibit distended lysosomes (Aula et al., 1979; Goldin et al., 1995; Malm and Nilssen, 2008; Arvio and Mononen, 2016), suggesting that they are under high hydrostatic pressure and membrane tension, which likely affects their trafficking. The connections between endomembrane tension and lysosomal trafficking in LSDs is unknown, but recent evidence would at least suggest a role in inward budding and outward tubulation(Freeman et al., 2020; Mercier et al., 2020). A thorough investigation of the osmotic pressure and endomembrane tension incurred in these LSDs would greatly contribute to our understanding of the disease pathology.

#### The Role of Endomembrane Tension in Infection

Membrane tension is potentially targetable. The first clues come from studies of infections caused by the bacterium *Helicobacter pylori* that colonizes the gastric mucosa, and can cause chronic inflammation, stomach ulcers, and gastric cancer (Suerbaum and Michetti, 2002). This bacterium survives by entering the endolysosomal system, and causes extreme vacuolation of the late endosomes/lysosomes within which it resides (Leunk et al., 1988; Amieva et al., 2002). The lysosomal vacuolation is largely attributed to a toxin, VacA, produced by the bacterium (Terebiznik et al., 2006), that has been classified as a pore-forming channel able to conduct Cl^−^, bicarbonate and small organic anions (Foegeding et al., 2016). Interestingly, TRPML activity is reduced in infected cells and activation of TRPML prevents the VacA-induced lysosomal swelling (Capurro et al., 2019). These findings suggest that the disruption of solute transport causes osmotic swelling of the *H. pylori* vacuole that is likely under high membrane tension. Many bacteria have evolved ways to hijack the host trafficking machinery in order to survive in intracellular compartments (Cossart and Helenius, 2014). *Listeria monocytogenes* and *Chlamydia trachomatis* for example are also known to cause spacious vacuoles to form that accommodate their infection cycle (Van Ooij et al., 1997; Birmingham et al., 2008). In the case of *H. pylori, L. monocytogenes* and C. *trachomatis*, manipulation of host endomembrane tension may be a mechanism of arresting traffic to prevent resolution of their niche.

As control of membrane tension is required for membrane bending, it is also not surprising that pathogens that require fusion with host endomembranes are opposed by high hydrostatic pressure and membrane tension. Enveloped viruses that gain entry to the host cytosol by fusing with the (endo)lysosome including Ebola and coronaviruses do so with the use of fusogenic peptides, splayed by proteolytic enzyme processing (White et al., 2008). The fusogens must overcome the glycocalyx of the endosome for their insertion and the hydration force of two opposing bilayers; tension on the host membrane arrests the fusion process altogether (Harrison, 2008; Mercer et al., 2020). In this regard, it is interesting that PIKfyve and TPC inhibitors that prevent the entry of at least some enveloped viruses have been suggested as therapeutics (Kang et al., 2020). The treatment of human macrophages with TPC inhibitors blocks Ebola virus entry and the drug is effective *in vivo* to prevent infection in mice (Sakurai et al., 2015). So, while understanding endomembrane tension in cellular homeostasis is critical to appreciate normal trafficking events, it may also be important from a translational perspective.

## Conclusion

Studies on membrane trafficking typically focus on cargo (receptors and ligands) sorting and membrane remodeling. However, it is important also to consider the fate of the fluid that is taken up during the course of all endocytic uptake mechanisms. So often we neglect the “black space” of the endocytic pathway. We now know that fluid resolution by solute transport mechanisms is a critical prerequisite for early endosomal trafficking. At the lysosome, a complex balance between enzymatic digestion and solute efflux ensures that membrane tension is controlled to permit lysosomal trafficking. When solute transport is impaired, this balance is perturbed, resulting in numerous LSDs. In addition, endomembrane tension may be exploited in infection.

As we first introduced, membrane tension is a biophysical feature of membranes that has been well-appreciated for the PM and during endocytosis in particular (Popescu et al., 2006; El Alaoui Faris et al., 2009; Dent et al., 2016; Colom et al., 2018). Though we did not include a discussion of the extensive work exploring the impact of tension on reconstituted systems including bilayers and giant unilamellar vesicles (GUVs) in this article, tension has indeed been measured in such experimental set-ups and we direct the reader to the review by Kozlov and Chernomordik for a summary of these details (Kozlov and Chernomordik, 2015). Measurements of endolysosomal membrane tension in particular have been challenging due to numerous limitations. First, these organelles are small, and many are below the resolution of light microscopy. Second, endolysosomes are dynamic organelles that undergo frequent bi-directional transport and membrane remodeling, as described. Third, all biological membranes are subject to continuous changes, such as in the lipid species present, due to ongoing biochemical reactions. Nonetheless, fluorescent probes that intercalate into membranes and display changes in their photophysical properties in response to membrane tension, show promise for endomembrane tension measurement (Colom et al., 2018).

More work is needed to uncover the mechanisms cells use to sense endomembrane tension. For now, at least, there is ample evidence that cells use ion transport as a mechanism to control membrane tension to direct endolysosomal trafficking. Placing priority on understanding how these mechanisms are controlled in space and time would seem to be the logical next step.

## Author Contributions

AS and SAF wrote and reviewed the manuscript. Both authors contributed to the article and approved the submitted version.

## Conflict of Interest

The authors declare that the research was conducted in the absence of any commercial or financial relationships that could be construed as a potential conflict of interest.
